# Protective Effect of Casperome^®^, an Orally Bioavailable Frankincense Extract, on Lipopolysaccharide- Induced Systemic Inflammation in Mice

**DOI:** 10.3389/fphar.2018.00387

**Published:** 2018-04-20

**Authors:** Konstantin Loeser, Semjon Seemann, Stefanie König, Isabell Lenhardt, Mona Abdel-Tawab, Andreas Koeberle, Oliver Werz, Amelie Lupp

**Affiliations:** ^1^Institute of Pharmacology and Toxicology, Jena University Hospital, Jena, Germany; ^2^Chair of Pharmaceutical Medicinal Chemistry, Institute of Pharmacy, Friedrich Schiller University Jena, Jena, Germany; ^3^Central Laboratory of German Pharmacists, Eschborn, Germany

**Keywords:** frankincense, lipopolysaccharides, systemic inflammation, cytokines, oxidative stress, liver function

## Abstract

**Introduction:** Despite recent advances in critical care, sepsis remains a crucial cause of morbidity and mortality in intensive care units. Therefore, the identification of new therapeutic strategies is of great importance. Since ancient times, frankincense is used in traditional medicine for the treatment of chronic inflammatory disorders such as rheumatoid arthritis. Thus, the present study intends to evaluate if Casperome^®^ (Casp), an orally bioavailable soy lecithin-based formulation of standardized frankincense extract, is able to ameliorate systemic effects and organ damages induced by severe systemic inflammation using a murine model of sepsis, i.e., intraperitoneal administration of lipopolysaccharides (LPS).

**Methods:** Male 60-day-old mice were assigned to six treatment groups: (1) control, (2) LPS, (3) soy lecithin (blank lecithin without frankincense extract), (4) Casp, (5) soy lecithin plus LPS, or (6) Casp plus LPS. Soy lecithin and Casp were given 3 h prior to LPS treatment; 24 h after LPS administration, animals were sacrificed and health status and serum cytokine levels were evaluated. Additionally, parameters representing liver damage or liver function and indicating oxidative stress in different organs were determined. Furthermore, markers for apoptosis and immune cell redistribution were assessed by immunohistochemistry in liver and spleen.

**Results:** LPS treatment caused a decrease in body temperature, blood glucose levels, liver glycogen content, and biotransformation capacity along with an increase in serum cytokine levels and oxidative stress in various organs. Additionally, apoptotic processes were increased in spleen besides a pronounced immune cell infiltration in both liver and spleen. Pretreatment with Casp significantly improved health status, blood glucose values, and body temperature of the animals, while serum levels of pro-inflammatory cytokines and oxidative stress in all organs tested were significantly diminished. Finally, apoptotic processes in spleen, liver glycogen loss, and immune cell infiltration in liver and spleen were distinctly reduced. Casp also appears to induce various cytochromeP450 isoforms, thus causing re-establishment of liver biotransformation capacity in LPS-treated mice.

**Conclusion:** Casp displayed anti-inflammatory, anti-oxidative, and hepatoprotective effects. Thus, orally bioavailable frankincense extracts may serve as a new supportive treatment option in acute systemic inflammation and accompanied liver dysfunction.

## Introduction

Despite recent advances in critical care, sepsis, septic shock, and subsequent multi-organ failure remain an important cause of morbidity and mortality in intensive care units (Engel et al., 2007; Martin, 2012; Gaieski et al., 2013). About 20–25% of patients with sepsis and associated organ failure display early impaired liver function, which in severe cases can result in acute liver failure (Kramer et al., 2007; Koch et al., 2011; Nesseler et al., 2012; Horvatits et al., 2013; Yan et al., 2014). Such liver dysfunction seems to be mediated to a considerable extent by pro-inflammatory cytokines such as TNF-α, IL-6, and IL-8 that are liberated by resident macrophages of the liver, the so-called Kupffer cells (Nesseler et al., 2012; Yan et al., 2014). Moreover, it has been shown that the severity of liver damage (even more than the impairment of lung or kidney function) is of major consequence for overall patient outcome (Kramer et al., 2007; Koch et al., 2011; Nesseler et al., 2012; Horvatits et al., 2013; Yan et al., 2014). However, although extensive research efforts have been made, no specific therapy exists so far for the treatment of sepsis and of sepsis-associated liver dysfunction (Nesseler et al., 2012; Horvatits et al., 2013). Therefore, the identification of new therapeutic strategies is of great clinical importance (Yan et al., 2014).

Extracts of frankincense gum resins from different *Boswellia* species have been used for centuries in traditional medicine, e.g., in Indian Ayurveda medicine, in China, and in several African countries, to treat infectious diseases and chronic inflammatory disorders. But also in Europe, frankincense extracts are commonly and increasingly used, and several clinical trials have been conducted to demonstrate their therapeutic benefit (Abdel-Tawab et al., 2011). Recent data from both animal experiments and clinical trials revealed that gum resin extracts from *Boswellia* species not only exert beneficial effects in various inflammatory conditions such as, e.g., rheumatoid arthritis, osteoarthritis, ulcerative colitis, Crohn’s disease, multiple sclerosis, or bronchial asthma, but also in hyperlipidemia, obesity, and cancer (see e.g., Poeckel and Werz, 2006; Moussaieff and Mechoulam, 2009; Abdel-Tawab et al., 2011; Du et al., 2015; Stürner et al., 2017). Additionally, in 2002 the European Medicines Agency classified *Boswellia serrata* extract as an “orphan drug” for the treatment of peritumoral brain edema. The main active principles of these extracts are pentacyclic triterpenes, termed boswellic acids, which are unique to *Boswellia* species (Ammon, 2006). Among others, there are four important β-configurated boswellic acid derivatives: 3-*O*-acetyl-11-keto-β-boswellic acid (AKBA), 3-*O*-acetyl-β-boswellic acid (AβBA), 11-keto-β-boswellic acid (KBA), and β-boswellic acid (β-BA).

The exact molecular basis for the pharmacological actions of boswellic acids in chronic inflammatory conditions still remains to be resolved. As possible target structures microsomal prostaglandin E_2_ synthase-1, 5-lipoxygenase, human leukocyte elastase, cathepsin G, topoisomerase I and II, as well as members of the IκB kinase/NFκB and MAPK signaling pathways have been identified (Syrovets et al., 2005; Poeckel and Werz, 2006; Takada et al., 2006; Cuaz-Pérolin et al., 2008; Moussaieff and Mechoulam, 2009; Siemoneit et al., 2009, 2011; Ammon, 2010; Abdel-Tawab et al., 2011). Additionally, using precipitation experiments with immobilized boswellic acids, LPS has recently been identified as a possible direct molecular interaction partner of distinct boswellic acids and their synthetic derivatives (Henkel et al., 2012). The authors were also able to show that distinct boswellic acids are able to inhibit LPS-mediated effects in a series of cell-based assays via direct binding to LPS. The experiments revealed clear-cut structure–activity relationships and β-BA was found to be the most potent derivative. These findings are further supported by the fact that boswellic acids as well as frankincense resin extracts reduce LPS-induced NO and proinflammatory cytokine production in macrophages both *in vitro* and *in vivo* (Syrovets et al., 2000; Pandey et al., 2005; Gayathri et al., 2007; Sharma et al., 2016), thereby exerting hepato- and renoprotective effects (Pandey et al., 2005).

To the best of our knowledge, neither individual boswellic acids nor frankincense extracts have been evaluated for their effects in acute systemic inflammatory conditions before. Therefore, the aim of the present study was to investigate for the first time the impact of Casp, an orally bioavailable soy lecithin-based delivery form of standardized frankincense extract, in a murine model of acute systemic inflammation, i.e., the intraperitoneal administration of LPS. The LPS model was chosen based on the assumption of a direct interaction between boswellic acids, especially β-BA and LPS (Henkel et al., 2012). Additionally, in comparison to other sepsis animal models such as peritoneal contamination and infection with (human) stool bacteria (PCI) or cecal ligation and puncture (CLP), the LPS model has several essential advantages such as technical ease and high reproducibility (Seemann et al., 2017; Stortz et al., 2017). Furthermore, the LPS model has been proven to be most suitable for studying effects of new therapies on acute systemic inflammation (Seemann et al., 2017). Casp is composed of *Boswellia serrata* extract and soy lecithin in a 1:1 ratio, with about half part of microcrystalline cellulose added to improve physical stability. The formulation is standardized to contain ≥25% of triterpenoic acids by HPLC. Compared with poorly bioavailable non-formulated frankincense extracts, Casp provides a quicker absorption as well as considerably higher plasma and tissue levels of the most important boswellic acids, including β-BA (Hüsch et al., 2013; Riva et al., 2016). Since in rats maximum plasma levels were reached after 180 min (Hüsch et al., 2013), in the present study Casp was administered to the mice 3 h before LPS challenge; 24 h after LPS administration, animals of the different treatment groups (soy lecithin alone; Casp alone; soy lecithin plus LPS; Casp plus LPS) were evaluated in comparison to control mice and to LPS-only-treated animals with regard to general condition, body temperature, blood glucose values, and serum cytokine concentrations. Additionally, parameters of oxidative stress were determined in different organs and immune cell distribution in two organs decisively involved in sepsis pathogenesis, the liver and spleen. Since liver function is of importance for overall patient outcome, liver glycogen content and biotransformation capacity, representing two important liver function parameters, were also assessed. Finally, to further substantiate the animal studies, cell-based experiments using human peripheral blood mononuclear cells (PBMCs) were performed to confirm the suppressive effect of Casp on cytokine release *in vitro*.

## Materials and Methods

### Animals and Experimental Procedure

The study was conducted under the license of the Thuringian Animal Protection Committee (approval number: 02-011/12). The principles of laboratory animal care and the German Law on the Protection of Animals as well as the Directive 2010/63/EU were followed. Male adult C57BL/6N mice (60-days-old, b.wt. 25–30 g; Charles River Laboratories, Sulzfeld, Germany) were used and the animals were housed in plastic cages under standardized conditions (light–dark cycle 12/12 h, temperature 22 ± 2°C, humidity 50 ± 10%, pellet diet Altromin 1316, water *ad libitum*).

A total of 48 mice were randomly divided into six groups (*n* = 8 each): control (vehicle-treated animals; 0.9% NaCl, used for dissolution of LPS), LPS, soy lecithin, Casp, soy lecithin plus LPS, and Casp plus LPS. LPS (*Escherichia coli* 0111:B4, Sigma-Aldrich, Steinheim, Germany) was dissolved in 0.9% NaCl and injected intraperitoneally at a dosage of 5 mg/kg b.wt. With this dosage, a medium-grade non-lethal systemic inflammation was intended to allow for detection of possible beneficial effects of co-administered drugs (Seemann and Lupp, 2015, 2016; Seemann et al., 2017). Thus, no antibiotic treatment or fluid resuscitation of the animals was necessary. Soy lecithin and Casp (kindly donated by Indena S.p.A., Milano, Italy) were given orally at a dosage of 80 mg/kg b.wt. (equivalent to the proportion of soy lecithin in Casp) or 240 mg/kg b.wt., respectively, 3 h before LPS challenge to ensure maximum plasma levels of boswellic acids at the time of LPS challenge (Hüsch et al., 2013). Based on previous findings (Seemann et al., 2017), animals were sacrificed 24 h after LPS challenge when plasma levels of pro-inflammatory cytokines are still elevated and changes in immune cell distribution and liver function are already detectable. Twenty-four hours after LPS treatment, body temperature was measured and the mice’s health status was assessed by using the CSS described previously (Gonnert et al., 2011). Afterward, mice were sacrificed with an overdose of isoflurane, decapitated, and bled completely. Whole blood was collected in a tube (S Monovette^®^ 1.2 ml Z Clotting Activator/Serum, Sarstedt, Nümbrecht, Germany) for clotting. Blood glucose levels were determined using a droplet of the whole blood with a commercially available blood glucose meter and respective test strips (BG Star^®^, Sanofi-Aventis, Frankfurt, Germany). After 30 min, clotted blood was centrifuged at 2000 × *g* for 10 min to obtain serum which was used for ELISA and enzymatic activity measurements. Additionally, brain, thymus, heart, lung, spleen, kidneys, liver, and adrenals were removed, weighed, and either fixed in 10% buffered formaldehyde or snap-frozen in liquid nitrogen for biochemical analysis. For histological analyses, formalin-fixed organ samples were embedded in paraffin blocks (*n* = 8 animals for each treatment group).

### Interleukin (IL)-6, IL-10, Tumor Necrosis Factor (TNF)-α, and Alanine Aminotransferase (ALAT) Assays

Serum levels of inflammatory and liver damage markers were quantified with the following commercially available reagents: mouse TNF-α Quantikine ELISA Kit (R&D Systems, Minneapolis, MN, United States), IL-6 Mouse ELISA Kit (Thermo Scientific, Rockford, IL, United States), IL-10 Mouse ELISA Kit (Thermo Scientific), and EnzyChrom^TM^ Alanine Transaminase Assay Kit (BioAssay Systems, Hayward, CA, United States).

### Oxidative Status in the Tissues

To determine tissue content of GSH and GSSG, samples were homogenized with 11 volumes of 0.2 M sodium phosphate buffer [5 mM ethylenediaminetetraacetic acid (EDTA); pH 8.0] and 4 volumes of 25% metaphosphoric acid. After centrifugation (12,000 × *g*, 4°C, 30 min), GSH was measured photometrically in the supernatants (Ellman, 1959) and GSSG fluorometrically (Hissin and Hilf, 1976). To assess the tissue content of LPO as TBARS, tissue samples were homogenized with 19 volumes of ice-cold saline and analyzed fluorometrically, as previously described (Yagi, 1987).

### Liver Biotransformation Capacity

Biotransformation capacity was assessed in the 9000 × *g* supernatants of the livers. To obtain 9000 × *g* supernatants, livers were homogenized with 0.1 M sodium phosphate buffer (pH 7.4) (1:3, w/v) and subsequently centrifuged at 9000 × *g* for 20 min at 4°C. The protein content of these fractions was determined using a modified Biuret method (Klinger and Müller, 1974). For determination of CYP enzyme activities, the following model reactions for different CYP isoforms were performed: ECOD [CYP1A, 2A, 2B, 2C, 2E (Aitio, 1978)], EROD [CYP1A (Pohl and Fouts, 1980)], MROD [CYP1A2 (Lubet et al., 1985)], PROD [CYP2B (Lubet et al., 1985)], BROD [CYP2A, 2B, 2C, 3A (Lubet et al., 1985)], and EMND [CYP3A (Kleeberg and Klinger, 1982)]. The amount of metabolite formed in these model reactions was normalized to the protein content of the respective 9000 × *g* supernatants and expressed as (pmol × mg protein^-1^ × min^-1^).

### Histopathology and Immunohistochemistry

Samples for histopathology and immunohistochemistry were prepared by cutting 4-μm sections from the paraffin blocks and floating these onto positively charged slides. Immunostaining was performed by an indirect peroxidase-labeling method, as described previously (Kaemmerer et al., 2017). Briefly, sections were de-waxed, microwaved in 10 mM citric acid (pH 6.0) for 16 min at 600 W, and incubated with the respective primary antibodies (**Table 1**) at 4°C overnight. Detection of the primary antibodies was performed using either biotinylated goat anti-rabbit, horse anti-mouse or rabbit anti-goat IgGs, followed by incubation with peroxidase-conjugated avidin (Vector ABC “Elite” Kit, Vector, Burlingame, CA, United States). Binding of the primary antibody was visualized using 3-amino-9-ethylcarbazole (AEC) in acetate buffer (BioGenex, San Ramon, CA, United States). Sections were then rinsed, counterstained with Mayer’s hematoxylin (Sigma-Aldrich, Steinheim, Germany), and mounted in VectaMount^TM^ Mounting Medium (Vector Laboratories, Burlingame, CA, United States). All immunohistochemical stainings were evaluated by two independent blinded investigators. To detect liver glycogen content, periodic-acid-Schiff staining (PAS; periodic acid, Schiff’s reagent: Sigma-Aldrich, Steinheim, Germany) was performed, and to obtain a histological overview, hematoxylin and eosin staining (HE) of livers and spleens was conducted, using standard protocols (McManus, 1948; Fischer et al., 2008). Identification of the specific cell types was based on their microscopic features along with the relative location of the cells in the respective tissues.

**Table 1:** Primary antibodies used for immunohistochemistry.

Antibody	Type, Catalog-No.	Manufacturer	Dilution	Host
CD3	Monoclonal, ab16669	Abcam	1:400	Rabbit
CD68	Monoclonal, ab955	Abcam	1:500	Mouse
Cleaved caspase-3	Monoclonal, 9661	Cell Signaling Technology	1:600	Rabbit
CYP1A	Polyclonal	Daiichi Pure Chemicals	1:5000	Goat
CYP2B	Polyclonal	Daiichi Pure Chemicals	1:5000	Goat
CYP3A	Polyclonal	Daiichi Pure Chemicals	1:5000	Goat
F4/80	Monoclonal, MCA497G	Bio-Rad Laboratories	1:200	Rat
iNOS	Polyclonal, sc-651	Santa Cruz Biotechnology	1:500	Rabbit
TNF-α	Monoclonal, sc-52746	Santa Cruz Biotechnology	1:500	Mouse

### Cell Culture Experiments

#### Cells

Peripheral blood (Institute for Transfusion Medicine, Jena University Hospital, Jena, Germany) was collected from healthy adult donors who had not taken any anti-inflammatory drugs during the last 10 days. The protocols for experiments with human monocytes were approved by the ethical committee of the Friedrich Schiller University Jena, Jena, Germany, on March 19, 2014; approval number: 4025-02/14. All methods were performed in accordance with the relevant guidelines and regulations. Leukocyte concentrates were centrifuged (4000 × *g*, 20 min, 20°C) and PBMCs were freshly isolated by dextran sedimentation and centrifugation on lymphocyte separation medium (Histopaque^®^-1077, Sigma-Aldrich, Steinheim, Germany). Resulting PBMCs were seeded in RPMI 1640 (Sigma-Aldrich, Steinheim, Germany) supplemented with 10% heat inactivated FCS, 100 U/ml penicillin, and 100 μg/ml streptomycin in cell culture flasks (Greiner Bio-one, Nuertingen, Germany) for 1–1.5 h at 37°C, 5% CO_2_. Adherent monocytes were washed twice with PBS and finally harvested by cell scraping.

#### Cytokine Measurement

In order to determine the effect of Casp on cytokine release, 1.5 × 10^6^ monocytes/ml in RPMI 1640 (supplemented with 5% heat inactivated FCS, 100 U/ml penicillin, and 100 μg/ml streptomycin) were pre-incubated for 30 min with vehicle (0.2% DMSO) or 30 μg/ml Casp after allowing for monocyte adhesion for 1.5 h at 37°C and 5% CO_2_. Cells were stimulated with 10 ng/ml LPS for distinct intervals (TNF-α, IL-8: 4 h; IL-6, IL-10: 18 h), and cytokines released into the medium were measured using specific Quantikine ELISA Kits (R&D Systems, Minneapolis, MN, United States). The glucocorticoid dexamethasone (1 μM; Sigma-Aldrich, Steinheim, Germany) was used as positive control.

#### MTT Assay

To exclude cytotoxic effects of Casp, monocytes were diluted to 2 × 10^6^/ml in RPMI 1640 (containing 5% heat inactivated FCS, 100 U/ml penicillin, and 100 μg/ml streptomycin) and seeded in a 96-well plate. Monocytes were allowed to adhere for 1.5 h at 37°C and 5% CO_2_ prior to treatment with vehicle (0.5% DMSO) or extract (30 μg/ml) in 100 μl of medium for 24 h. Afterward, cells were incubated with thiazolyl blue tetrazolium bromide solution (MTT, 5 mg/ml PBS; Sigma-Aldrich, Steinheim, Germany) until blue staining of the control. Formazan formation was stopped by adding 100 μl SDS-lysis buffer (10%, w/v in 20 mM HCl, pH 4.5) and the well plates were shaken at room temperature overnight. Finally, absorbance was measured at 570 nm with a Multiskan^TM^ microplate spectrophotometer (Thermo Scientific, Ulm, Germany). The pan-protein kinase inhibitor staurosporine (1 μM; Calbiochem, La Jolla, CA, United States) was used as positive (cytotoxic) control.

### Statistical Analysis

For statistical analysis, the IBM SPSS statistics program (version 22.0) was used. In all cases, animal experiments were performed with eight mice per treatment group. Statistical significance was determined by using the non-parametric Kruskal–Wallis test, followed by the Mann–Whitney *U*-test with Holm–Bonferroni correction. A *p*-value ≤ 0.05 was considered statistically significant. Data are given as mean ± standard error of the mean (SEM).

Cell culture experiments were conducted in triplicate. Data are given as means ± SEM. Statistically significant differences in comparison to LPS-stimulated control cells were determined by one-way ANOVA plus Bonferroni *post hoc* test and are denoted as follows: ^∗^*p* ≤ 0.05; ^∗∗^*p* ≤ 0.01; ^∗∗∗^*p* ≤ 0.001.

## Results

### Health Status, Body Temperature, and Blood Glucose Values

**FIGURE 1:**
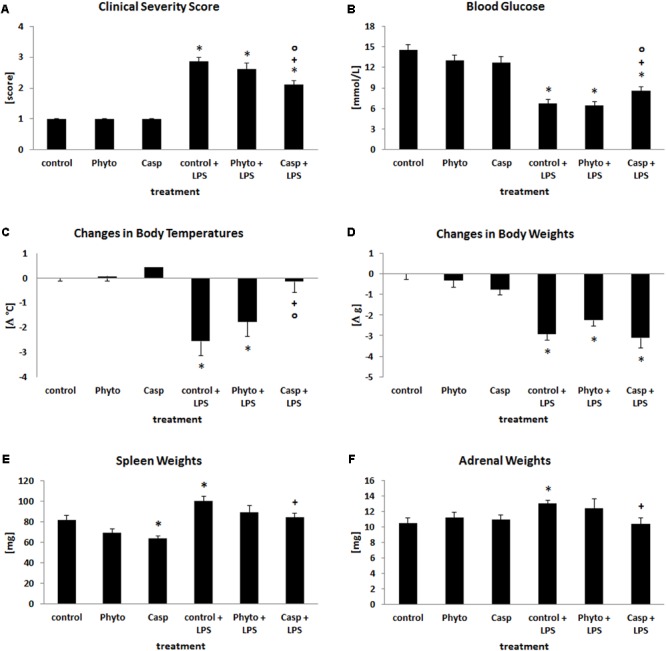
Clinical severity score **(A)**, blood glucose values **(B)**, changes in body temperatures **(C)**, changes in b.wt. **(D)**, spleen **(E)**, and adrenal weights **(F)**. Mice were administered either vehicle (control), soy lecithin (Phyto), Casp, LPS (control + LPS), soy lecithin + LPS, or Casp plus LPS (Casp + LPS). Data are given as means ± SEM, *n* = 8 for each group. ^∗^, significantly different from controls; +, significantly different from LPS-treated animals; o, significantly different from soy lecithin plus LPS-treated animals (*p* ≤ 0.05; Mann–Whitney *U*-test followed by Holm–Bonferroni correction).

To examine the systemic influence of Casp on LPS-induced systemic inflammation, health status as assessed by the CSS, body temperature, blood sugar level, and body and organ weight was determined. Twenty-four hours after treatment, the health status of LPS-challenged mice was distinctly impaired (**Figure 1A**). Additionally, body temperature was significantly decreased by an average of 2.6°C (mean control value: 37.2°C). Blood glucose levels were even diminished by more than 50% when compared with control animals (**Figures 1B,C**). In all cases, Casp was able to significantly ameliorate LPS-induced effects, whereas soy lecithin (solvent control, devoid of frankincense) showed no protective impact. Mice receiving soy lecithin or Casp alone displayed no relevant differences to the control group.

In comparison to controls, LPS challenge caused an average decrease in b.wt. by 14% (**Figure 1D**) and in liver and thymus weights by 12 and 25%, respectively, whereas spleen and adrenal weights were increased by 23% (**Figures 1E,F**). Weights of brain, kidney, and heart, in contrast, remained unaffected (data not shown). Treatment with soy lecithin or Casp alone caused a decrease in spleen weights by 15 and 22% (*p* = 0.071; *p* = 0.007) and in thymus weights by 21 and 29% (*p* = 0.066; *p* = 0.016), respectively (**Figure 1E**), with no overt histological changes in these organs. While administration of soy lecithin had no effect on LPS-mediated changes in body and organ weights, Casp was able to significantly return the LPS-induced increase in spleen and adrenal weights to control values (**Figures 1E,F**). Thymus weights, in contrast, remained significantly reduced not only by additional soy lecithin treatment, but also by co-administration of Casp by 39 and 34% (*p* = 0.006; *p* = 0.007), respectively, which was not statistically different from the weight reduction observed after sole LPS treatment (*p* = 0.167; *p* = 0.264; data not shown).

### Serum Cytokine and ALAT Levels

**FIGURE 2:**
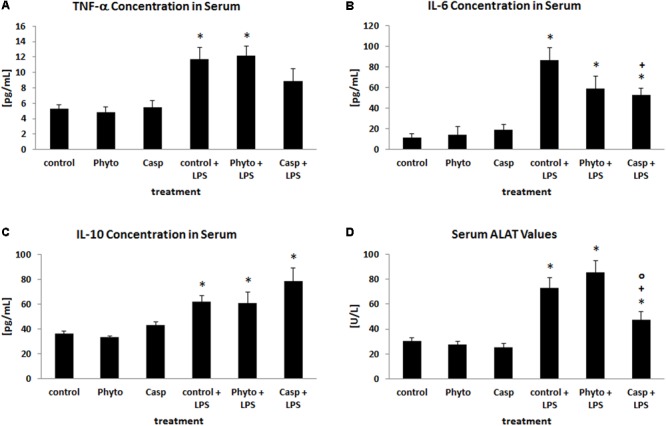
Serum concentrations of TNF-α **(A)**, IL-6 **(B)**, IL-10 **(C)**, and ALAT **(D)**. Mice were administered either vehicle (control), soy lecithin (Phyto), Casp, LPS (control + LPS), soy lecithin + LPS, or Casp plus LPS (Casp + LPS). Data are given as means ± SEM, *n* = 8 for each group. ^∗^, significantly different from controls; +, significantly different from LPS-treated animals; o, significantly different from soy lecithin plus LPS-treated animals (*p* ≤ 0.05; Mann–Whitney *U*-test followed by Holm–Bonferroni correction).

To evaluate the impact of Casp on LPS-induced alterations in circulating cytokine levels, serum concentrations of the pro-inflammatory cytokines TNF-α and IL-6 as well as of the anti-inflammatory cytokine IL-10 were measured at sacrifice (**Figures 2A–C**). Compared to control animals, administration of LPS caused an elevation of serum TNF-α, IL-6, and IL-10 values by about 120, 670, and 70%, respectively, whereas sole treatment with soy lecithin or Casp had no effect. Co-administration of Casp was able to reduce the LPS-induced increase in serum TNF-α and IL-6 levels by about 30 and 50%, respectively, whereas IL-10 values were slightly further elevated. Co-administration of soy lecithin, in contrast, had no influence on LPS effects.

As a parameter indicating liver damage, serum ALAT levels were determined (**Figure 2D**). Here, LPS treatment caused a distinct increase by about 140% compared to controls. Sole soy lecithin or Casp administration, in contrast, had no influence on serum ALAT concentration and no effect of soy lecithin co-treatment on LPS-induced liver damage was noted. In contrast, after co-administration of Casp significantly lower ALAT values were observed compared to LPS-only-treated mice.

### Oxidative Stress in Different Organs

**FIGURE 3:**
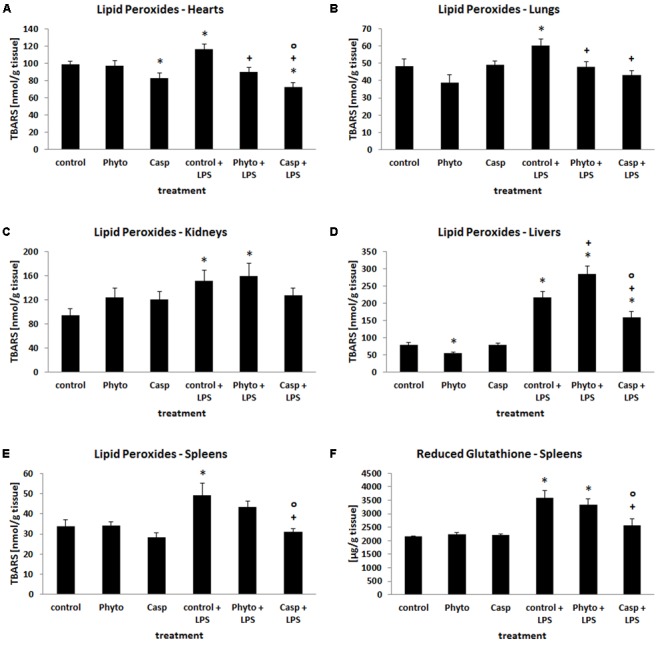
Tissue content of LPO in hearts **(A)**, lungs **(B)**, kidneys **(C)**, livers **(D)**, and spleens **(E)**, and concentration of GSH in spleen tissue **(F)** as a measure of oxidative stress in the organs. Mice were administered either vehicle (control), soy lecithin (Phyto), Casp, LPS (control + LPS), soy lecithin + LPS, or Casp plus LPS (Casp + LPS). Data are given as means ± SEM, *n* = 8 for each group. ^∗^, significantly different from controls; +, significantly different from LPS-treated animals; o, significantly different from soy lecithin plus LPS-treated animals (*p* ≤ 0.05; Mann–Whitney *U*-test followed by Holm–Bonferroni correction).

Since oxidative stress plays a major role in systemic inflammation, the impact of Casp on LPS-induced changes in the oxidative status was evaluated in different organs. For this purpose, tissue levels of lipid peroxidation products as well as the tissue content of GSH and GSSG were determined. After LPS challenge, tissue content of lipid peroxidation products was increased by 12, 20, 24, 45, 60, and 170% in brain, heart, lung, spleen, kidney, and liver, respectively, suggesting considerably elevated oxidative stress in these organs (**Figures 3A–E**). Soy lecithin administration alone caused a decrease in LPO levels only in liver by about 30%, compared to control levels. Similarly, only in heart LPO were decreased by about 20% after sole Casp treatment. Compared to LPS-only-treated animals, combined soy lecithin and LPS administration reduced LPO levels in lung, heart, and spleen, but increased the values in liver. In contrast, co-treatment with Casp significantly attenuated LPS-mediated elevation of LPO toward control in all organs investigated, thus indicating a noticeable additional antioxidant capacity of *Boswellia serrata* extract (**Figures 3A–E**). Regarding glutathione status, increases in GSH, GSSG, and total glutathione concentrations by about 10, 20, and 60% were noticed in kidney, lung, and spleen after LPS treatment (**Figure 3F**), respectively, while liver levels decreased by 10% (representing the primary organ of glutathione synthesis; data not shown). There was, however, no major change in the GSH/GSSG ratio in all tissues. In brain and heart, no influence of LPS administration on glutathione concentrations was observed. After additional administration of Casp to LPS-treated animals only in spleens a normalization of the glutathione status could be achieved (**Figure 3F**). In all other organs no relevant significant impact of either soy lecithin or Casp on LPS-mediated effects on tissue glutathione levels was observed.

### Spleen: Apoptosis, Immune Cell Redistribution, and TNF-α Expression

**FIGURE 4:**
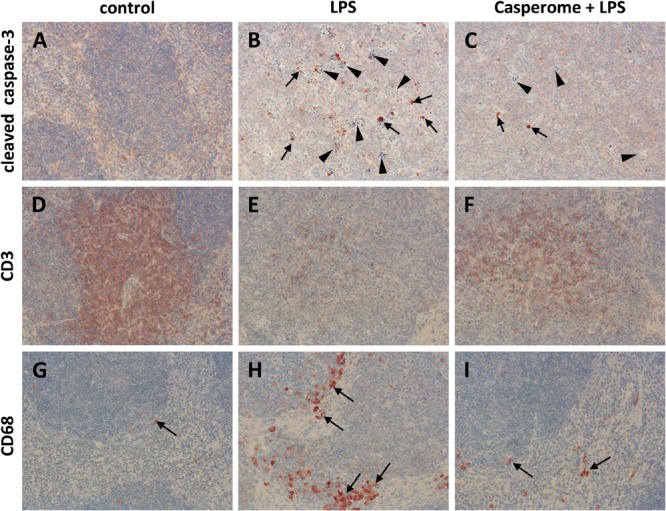
Cleaved caspase-3 **(A–C)**, CD3 **(D–F)**, and CD68 **(G–I)** expression in the spleens of control **(A,D,G)**, LPS **(B,E,H)**, or Casp plus LPS **(C,F,I)**-treated mice. Mice were administered either vehicle (control), soy lecithin, Casp, LPS (control + LPS), soy lecithin + LPS, or Casp plus LPS (Casp + LPS). Representative photomicrographs from one of eight different tissue samples from control mice, LPS-treated, and LPS plus Casp-treated animals are shown. Photomicrographs from soy lecithin- or Casp-only-treated mice and from soy lecithin plus LPS-treated mice are not depicted separately since they did not differ from controls or LPS-only-treated mice, respectively. Immunohistochemistry (red–brown color), counterstaining with hematoxylin; original magnification: ×400. Arrows in **B** and **C** mark cleaved caspase-3-positive apoptotic cells, arrowheads indicate tingible body macrophages; arrows in **G, H**, and **I** mark CD68-positive monocytes/macrophages.

**FIGURE 5:**
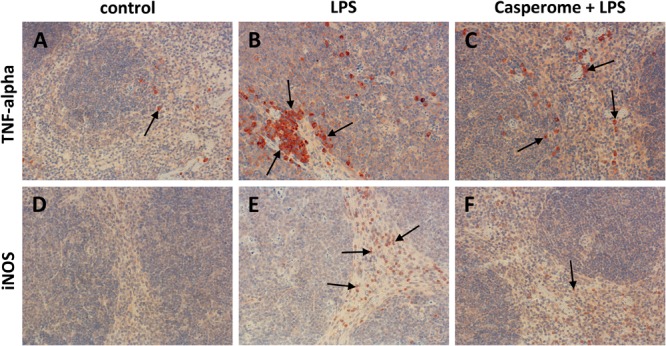
TNF-α **(A–C)** and iNOS **(D–F)** expression in the spleens of control **(A,D)**, LPS **(B,E)**, or Casp plus LPS **(C,F)**-treated mice. Mice were administered either vehicle (control), soy lecithin, Casp, LPS (control + LPS), soy lecithin + LPS, or Casp plus LPS (Casp + LPS). Representative photomicrographs from one of eight different tissue samples from control mice, LPS-treated, and LPS plus Casp-treated animals are shown. Photomicrographs from soy lecithin- or Casp-only-treated mice and soy lecithin plus LPS-treated mice are not depicted separately since they did not differ from controls or LPS-only-treated mice, respectively. Immunohistochemistry (red–brown color), counterstaining with hematoxylin; original magnification: ×400. Arrows in **A–C** mark TNF-α-positive monocytes/macrophages, arrows in **E** and **F** indicate iNOS-positive neutrophils, respectively.

**FIGURE 6:**
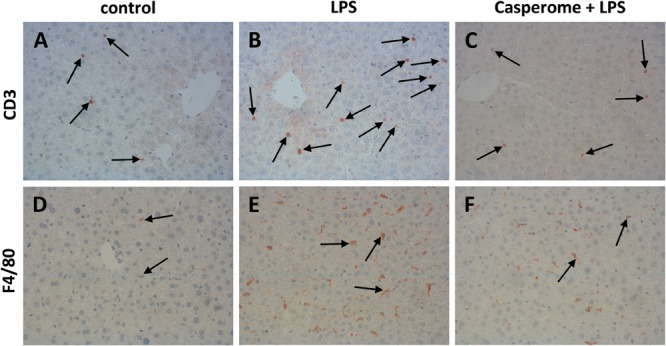
CD3 **(A–C)** and F4/80 **(D–F)** expression in the spleens of control **(A,D)**, LPS **(B,E)**, or Casp plus LPS **(C,F)**-treated mice. Mice were administered either vehicle (control), soy lecithin, Casp, LPS (control + LPS), soy lecithin + LPS, or Casp plus LPS (Casp + LPS). Representative photomicrographs from one of eight different tissue samples from control mice, LPS-treated, and LPS plus Casp-treated animals are shown. Photomicrographs from soy lecithin- or Casp-only-treated mice and soy lecithin plus LPS-treated mice are not depicted separately since they did not differ from controls or LPS-only-treated mice, respectively. Immunohistochemistry (red–brown color), counterstaining with hematoxylin; original magnification: ×400. Arrows in **A–C** mark CD3-positive T-lymphocytes, arrows in **D–F** indicate F4/80-positive macrophages, respectively.

**FIGURE 7:**
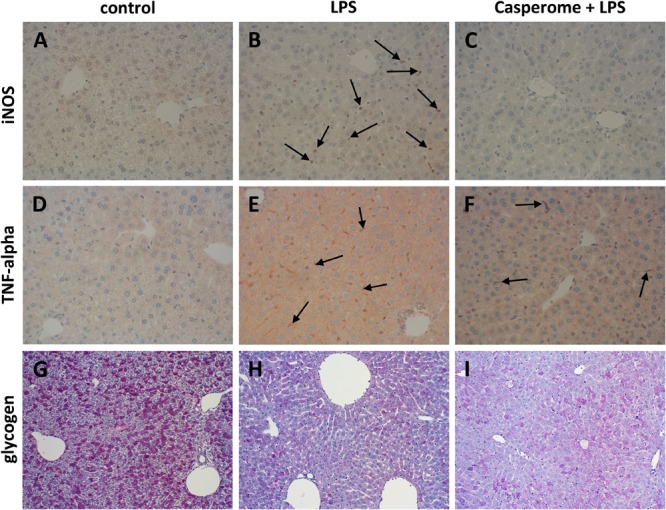
iNOS **(A–C)**, TNF-α expression **(D–F)**, and glycogen content **(G–I)** in the livers of control **(A,D,G)**, LPS **(B,E,H)**, or Casp plus LPS **(C,F,I)**-treated mice. Mice were administered either vehicle (control), soy lecithin, Casp, LPS (control + LPS), soy lecithin + LPS, or Casp plus LPS (Casp + LPS). Representative photomicrographs from one of eight different tissue samples from control mice, LPS-treated, and LPS plus Casp-treated animals are shown. Photomicrographs from soy lecithin- or Casp-only-treated mice and soy lecithin plus LPS-treated mice are not depicted separately since they did not differ from controls or LPS-only-treated mice, respectively. **A–F**: Immunohistochemistry (red–brown color), counterstaining with hematoxylin; **G–I**: periodic acid-Schiff staining; original magnification: ×400. Arrows in **B** mark iNOS-positive neutrophils, arrows in **E** and **F** indicate TNF-α-positive monocytes/macrophages.

**FIGURE 8:**
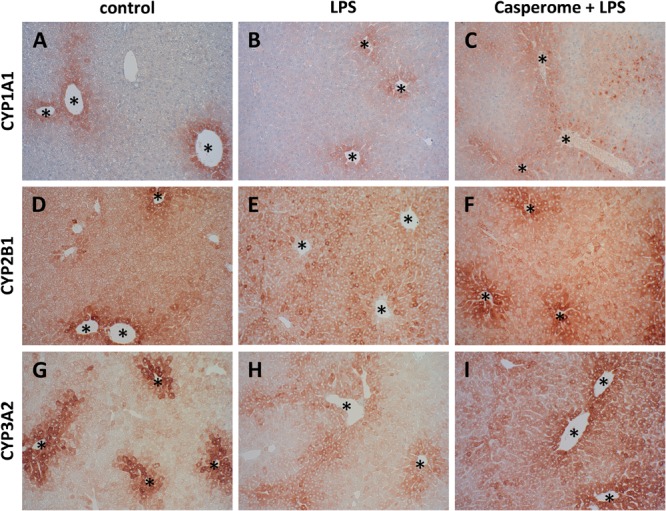
CYP 1A1 **(A–C)**, CYP2B1 **(D–F)**, and CYP3A2 **(G–I)** expression in the livers of control **(A,D,G)**, LPS **(B,E,H)**, or Casp plus LPS **(C,F,I)**-treated mice. Mice were administered either vehicle (control), soy lecithin, Casp, LPS (control + LPS), soy lecithin + LPS, or Casp plus LPS (Casp + LPS). Representative photomicrographs from one of eight different tissue samples from control mice, LPS-treated, and LPS plus Casp-treated animals are shown. Photomicrographs from soy lecithin- or Casp-only-treated mice and soy lecithin plus LPS-treated mice are not depicted separately since they did not differ from controls or LPS-only-treated mice, respectively. Immunohistochemistry (red–brown color), counterstaining with hematoxylin; original magnification: ×400. CYP expression was mainly confined to the hepatocytes around the central veins (asterisks).

Since apoptotic processes are of substantial importance in the course of systemic inflammation and spleen function is crucial for the removal of damaged red and white blood cells and of bacteria from the blood stream, cleaved caspase-3 expression was assessed in spleens by means of immunohistochemistry as a marker of apoptotic processes. Examination of spleens from LPS-only-treated mice revealed a markedly enhanced expression of cleaved caspase-3, especially in the white pulp, compared to controls and to soy lecithin^®^- or Casp-only-treated animals (**Figures 4A,B**). Besides being increasingly present in spleno- and lymphocytes, strong immunostaining was noticed especially in tingible body macrophages, which were much more frequently present in the white pulp of spleens from LPS-challenged mice than in the other treatment groups (arrowheads in **Figure 4B**). While no difference in cleaved caspase-3 expression was detected between LPS-only and soy lecithin-plus-LPS-treated mice, co-administration of Casp yielded a clear-cut reduction in cleaved-casepase-3 expression in spleen, thus indicating an anti-apoptotic effect (**Figure 4C**; NB: in **Figure 4** and **Figures 5–8** photomicrographs from soy lecithin- or Casp-only-treated mice and from soy lecithin plus LPS-treated mice are not depicted separately since they did not differ from controls or from LPS-only-treated mice, respectively).

To assess the effects of Casp on LPS-induced changes in immune cell distribution in the spleen, immunostainings for CD3 and CD68 as markers for T-lymphocytes and monocytes/macrophages, respectively, were performed. In control animals and in soy lecithin- or Casp-only-treated mice, CD3+ cells were abundantly present in the periarteriolar sheaths of the white pulp (**Figure 4D**). In contrast, after LPS challenge the splenic tissue was nearly devoid of CD3+ cells (**Figure 4E**). This effect was partially reversed after co-treatment with Casp (**Figure 4F**), but not with soy lecithin, indicating a specific immunomodulatory action of the frankincense extract. In contrast to the massive emigration of CD3+ positive cells from the splenic tissue after LPS administration, a considerable immigration of CD68+ cells was detected in spleens after LPS treatment of the animals when compared to controls or to soy lecithin- or Casp-only-treated mice (**Figures 4G,H**). These cells were mainly observed in the red pulp, but an increased infiltration of CD68+ cells was also noticed in the white pulp. Again, this effect was nearly completely reversed after co-administration of Casp, but not of soy lecithin (**Figure 4I**).

Tumor necrosis factor-α is mainly secreted by macrophages. Due to the massive immigration of CD68+ cells into the spleens after LPS treatment, TNF-α expression was additionally assessed in spleen tissue by immunohistochemistry. These investigations revealed TNF-α expression patterns, which were very similar to those seen in the CD68 immunostaining: In comparison to control or to soy lecithin- or Casp-only treatment, LPS-challenge caused a massive immigration of TNF-α-positive cells into the splenic tissue, which was almost completely abolished by co-treatment of the mice with Casp (**Figures 5A–C**), whereas co-administration of soy lecithin showed no effect.

Not only LPS, but also pro-inflammatory cytokines such as TNF-α are known to induce iNOS expression in immune cells such as neutrophils and monocytes/macrophages via the NFκB pathway. Besides giving rise to cytotoxic peroxynitrite, excess NO production is critically involved in the course of systemic inflammation, since it contributes to hypotension, cardiodepression, vascular hyporeactivity, and ultimately septic shock. Therefore, in the present investigation, iNOS expression was also assessed in spleen and liver tissue. Following LPS treatment, a massive immigration of iNOS-positive neutrophils (and occasionally also of macrophages) was detected in the red and occasionally also in the white pulp of the spleens from LPS-only-treated animals in comparison to control and soy lecithin- or Casp-only-treated mice (**Figures 5D,E**). Also here, co-administration of Casp, but not of soy lecithin, distinctly ameliorated this effect (**Figure 5F**).

### Liver: Immune Cell Immigration, Glycogen Content, and Biotransformation Capacity

In parallel to the investigations in the spleens, immunostainings for different immune cell markers and for TNF-α and iNOS were also performed in the livers. Here, in contrast to the spleens, an increased presence of CD3+ T-lymphocytes was observed after LPS treatment of the animals (**Figures 6A,B**). Additionally, liver expression of the macrophage marker F4/80 was distinctly higher in LPS-challenged mice in comparison to controls, soy lecithin- or Casp-only-treated animals (**Figures 6D,E**). Likewise, TNF-α expression was strongly enhanced in monocytes/macrophages and also in sinusoidal endothelial cells after LPS administration (**Figures 7D,E**). Additionally, and as in the spleens, LPS challenge yielded a massive appearance of iNOS-positive neutrophils in the livers (**Figures 7A,B**). All these effects were significantly less pronounced after pre-administration of Casp (**Figures 6C,F, 7C,F**), whereas soy lecithin had no impact on LPS-induced changes.

Since liver function is crucial for overall patient outcome in severe systemic inflammation, liver glycogen content and biotransformation capacity were assessed as representative parameters. In comparison to livers from control, soy lecithin- or Casp-only-treated mice, LPS-challenge caused almost complete glycogen depletion. This effect was partially reversed by additional administration of Casp but not of soy lecithin (**Figures 7G–I**).

**FIGURE 9:**
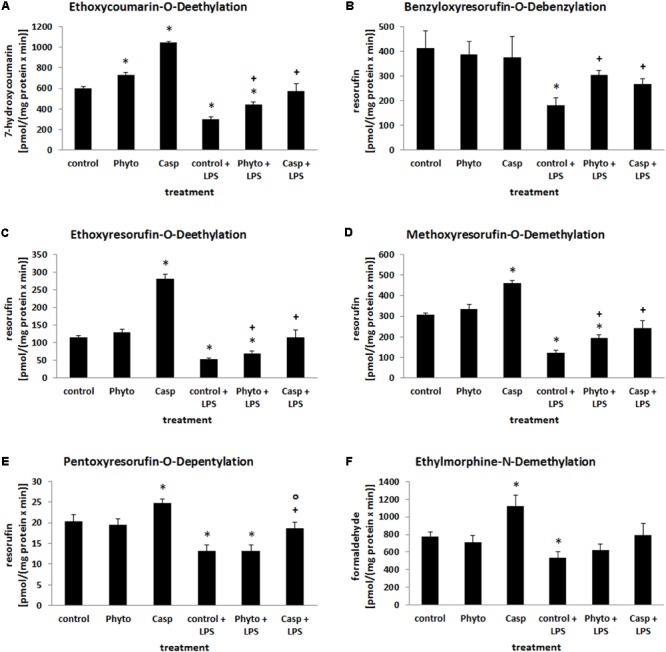
Biotransformation capacity as determined by a panel of model reactions for different CYP isoforms. Mice were administered either vehicle (control), soy lecithin (Phyto), Casp, LPS (control + LPS), soy lecithin + LPS, or Casp plus LPS (Casp + LPS). ECOD **(A)**, BROD **(B)**, EROD **(C)**, MROD **(D)**, PROD **(E)**, and EMND **(F)** were determined in the 9000 × *g* supernatants of the livers. Data are given as means ± SEM, *n* = 8 for each group. ^∗^, significantly different from controls; +, significantly different from LPS-treated animals; o, significantly different from soy lecithin plus LPS-treated animals (*p* ≤ 0.05; Mann–Whitney *U*-test followed by Holm–Bonferroni correction).

Biotransformation capacity was evaluated by assessing the expression levels of three different CYP isoforms and by measuring CYP-dependent monooxygenase activities by means of specific model reactions. All three CYP isoforms (CYP1A1, CYP2B1, and CYP3A2) assessed were predominantly expressed in the hepatocytes located around the central veins (**Figure 8**). In comparison to controls and soy lecithin-only-treated mice, LPS administration produced a strong reduction in CYP isoforms expression. In Casp-only-treated animals, in contrast, a strong increase in CYP expression was observed, which was most clearly visible in the pericentral regions of the liver lobules. Whereas co-administration of soy lecithin did not show any visible influence on LPS-mediated effects, additional treatment with Casp was able to restore CYP expression levels completely (**Figure 8**). Also in the CYP model reactions a distinct decrease in enzyme activities was observed after LPS administration in comparison to controls (**Figure 9**). In contrast, after sole soy lecithin and especially after sole Casp treatment distinct increases in CYP activities were observed, suggesting a noticeable CYP-inducing capacity of Casp (**Figure 9**). Whereas additional soy lecithin treatment in some instances slightly ameliorated the LPS effects, biotransformation capacity was completely restored back to control values after supplementary Casp administration (**Figure 9**).

### Effects of Casperome^®^ in Human Primary Monocytes

**FIGURE 10:**
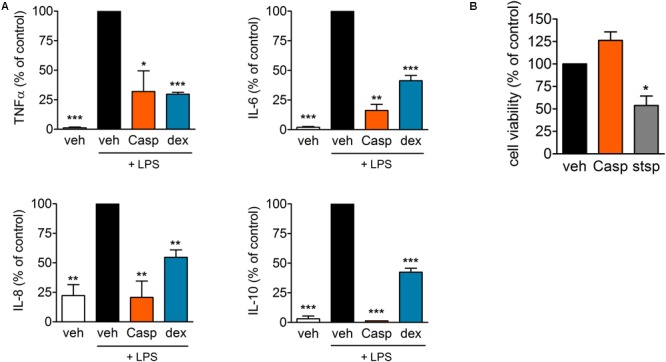
Effect of Casp on cytokine release and cytotoxicity in human monocytes. **(A)** Human primary monocytes were pre-incubated with vehicle or compounds (Casp; dexamethasone, dex) for 30 min prior to stimulation with LPS for 4 (TNF-α, IL-8) or 18 h (IL-6, IL-10). Formed cytokines were measured by ELISA. Data are means ± SEM; *n* = 3; ^∗∗∗^*p* < 0.001; ^∗∗^*p* < 0.01; ^∗^*p* < 0.05; inhibitor vs. stimulated control (100%), one-way ANOVA plus Bonferroni test. **(B)** Effect of Casp on cell viability. Intact human monocytes were incubated with compounds (Casp; staurosporine, stsp) or vehicle for 24 h and cell viability was analyzed by MTT assay. Data are means ± SEM; *n* = 3; ^∗^*p* < 0.05; inhibitor vs. vehicle control (100%), one-way ANOVA plus Bonferroni test.

In order to investigate anti-inflammatory effects of Casp in a human *in vitro* model, the influence of the extract on LPS-induced cytokine release from human monocytes was tested in comparison to the glucocorticoid dexamethasone. After pre-incubation with the test compounds, freshly isolated human primary monocytes were stimulated with 10 ng/ml LPS to elicit cytokine release (assessed by ELISA). At a concentration of 30 μg/ml, Casp decreased the levels of all pro-inflammatory cytokines measured. Thus, TNF-α release was reduced by 32% with similar efficiency as for dexamethasone (30% reduction). Even stronger effects of Casp were detected for release of IL-6 and the chemokine IL-8 (**Figure 10A**). Additionally, Casp and dexamethasone blocked the release of the anti-inflammatory cytokine IL-10. To exclude any cytotoxicity being responsible for diminished cytokine release, Casp was evaluated in a cell viability (MTT) assay. As shown in **Figure 10B**, 30 μg/ml Casp produced no detrimental impact on monocyte viability after 24 h incubation in comparison to control, whereas staurosporine reduced cell viability to about 54% of control values. Thus, these data support Casp as a potent anti-inflammatory agent without cytotoxic effects.

## Discussion

Since the beneficial effects of frankincense in the treatment of chronic inflammatory diseases have already been well documented (see e.g., Poeckel and Werz, 2006; Moussaieff and Mechoulam, 2009; Abdel-Tawab et al., 2011; Du et al., 2015), the aim of the present study was to assess possible protective effects of frankincense on acute systemic inflammation for the first time. Because of the well-documented influence of the boswellic acids on cytokine production by monocytes/macrophages (Syrovets et al., 2000; Pandey et al., 2005; Gayathri et al., 2007; Sharma et al., 2016), we focused on cytokine plasma levels, immune cell redistribution, and oxidative stress as well as liver function. The latter parameter is known to be critical for overall patient outcome during systemic inflammation and has been shown to be negatively influenced by pro-inflammatory cytokines (Morgan, 2001; Novotny et al., 2007; Jacob et al., 2009; Nesseler et al., 2012).

After LPS challenge, reduced b.wt. of the animals, decreased blood glucose levels, and almost complete glycogen depletion of the livers were observed, which is in line with literature data (Raetzsch et al., 2009; Santos et al., 2013; Seemann and Lupp, 2015, 2016; Seemann et al., 2017). On the one hand, these effects could be explained by the distinctly reduced health status of the animals, mirrored by low CSS scores, which may have led to reduced food intake. On the other hand, it has been shown that (partially mediated via increased TNF-α expression) LPS is able to reduce gluconeogenesis (Goto et al., 2001; Raetzsch et al., 2009; Santos et al., 2013) and to increase glycogenolysis (Casteleijn et al., 1988). Presumably by direct binding of the boswellic acids to LPS and thus blunting TLR4, MyD88, and NFκB signaling (and, consequently, TNF-α release and COX-2 induction), but probably also by a direct interaction with NFκB and COX-2, co-administration of Casp significantly increased blood glucose values and liver glycogen content compared to the LPS-only treatment group. Additionally, general health status of the animals was significantly improved after co-treatment with Casp with increased food consumption, lower levels of circulating pro-inflammatory cytokines, a lower severity of the inflammatory processes, and a normalization of the body temperatures, compared to the LPS-only-treated animals.

In the present investigation, markedly increased serum levels of the pro-inflammatory cytokines TNF-α and IL-6 were observed in LPS-treated animals, as expected from literature data (Seemann and Lupp, 2015, 2016; Seemann et al., 2017). These increased levels were distinctly reduced after Casp co-treatment of the mice. Thus, the reported suppression of pro-inflammatory cytokine release from monocytes/macrophages by boswellic acids (Syrovets et al., 2000; Pandey et al., 2005; Gayathri et al., 2007; Sharma et al., 2016) could be reproduced also with the frankincense extract Casp *in vivo* in acute systemic inflammation. These results were further confirmed by cell-based assays using human primary monocytes, which revealed a distinct reduction of the LPS-induced TNF-α, IL-6, and IL-8 release after Casp treatment. In contrast to the suppressive effects on pro-inflammatory cytokines, murine serum levels of the anti-inflammatory IL-10 were slightly enhanced by Casp co-treatment, whereas in the cell-based assay IL-10 release was strongly decreased. This discrepancy may be due to the fact that *in vivo* IL-10 is not only released by monocytes/macrophages but also by many other immune cells such as TH_2_- or Treg-lymphocytes. IL-10 released from these cells has a central role in limiting immune responses to pathogens to protect the organism from damage due to exaggerated inflammatory processes (Saraiva and O’Garra, 2010). Thus, in addition to the inhibitory effects seen on cytokine production by monocytes and macrophages, stimulation of IL-10 release from these other immune cells may have also contributed to the overall better outcome observed after co-treatment with Casp, compared to LPS-only-treated mice.

In parallel to the influence of Casp on pro-inflammatory and anti-inflammatory cytokines, also immune cell redistribution was distinctly reduced by the frankincense extract. In spleens, LPS caused a massive appearance of iNOS-positive neutrophils and of CD68/iNOS/TNF-α-positive monocytes/macrophages, but a depletion of CD3+ T-lymphocytes, whereas in livers immigration of iNOS positive neutrophils as well as CD3+ cells was observed. All these effects were distinctly ameliorated after co-administration of Casp. Especially the influence on iNOS expression may be of importance, since increased iNOS expression and, most importantly, immigration of iNOS-positive neutrophils, but also of iNOS-positive macrophages, into the tissues has been shown to be critical for the course of the disease and overall outcome (Kirkeboen and Strand, 1999; Tsukahara et al., 2001; Farley et al., 2006; Wang et al., 2012). Finally, in parallel to the positive influence on TNF-α serum values, Casp was also able to ameliorate LPS-induced increases in TNF-α expression by monocytes/macrophages and sinusoidal endothelial cells in liver tissue.

In contrast to average b.wt., spleen weights were distinctly enhanced after LPS treatment. The occurrence of splenomegaly in response to severe systemic inflammation has been shown also in humans (Arismendi-Morillo et al., 2004) and has been correlated with, among others, TNF-α- and IL-6-mediated elevation of serum high-mobility group box 1 (HMGB1) levels in spleen tissue (Valdés-Ferrer et al., 2013). The increase in volume has been demonstrated for both red and white pulp and has been shown to be due to hyperemia and enhanced immigration of white blood cells into the splenic tissue, especially neutrophils and macrophages (Valdés-Ferrer et al., 2013; Seemann et al., 2017). As mentioned above, an increased presence of iNOS-positive neutrophils and CD68/iNOS/TNF-α-positive monocytes/macrophages was observed in the spleens after LPS challenge also in the present investigation. Additionally, a distinct rise in apoptotic processes was also noted, demonstrated by increased cleaved caspase-3 stainings, and mirrored by increased presence of tingible body macrophages in spleens after LPS treatment. Probably, there is an increased need for degradation of nonfunctional erythrocytes and white blood cells as a result of systemic inflammation. In contrast to these processes, massive emigration of CD3+ T-lymphocytes from spleens was observed after LPS challenge, which is in line with previous investigations (Valdés-Ferrer et al., 2013; Seemann et al., 2017). Since the present investigation demonstrated increased immigration of CD3+ cells into liver tissue, these cells may leave the spleen to enter other organs in response to systemic inflammation. Probably by suppressing increased levels of pro-inflammatory cytokines, spleen weights were significantly decreased back to normal values after co-administration of Casp. Additionally, less neutrophils and macrophages were present in spleen tissue while apoptotic processes as well as presence of tingible body macrophages were distinctly reduced in comparison to LPS-only-treated animals. Furthermore, emigration of CD3+ cells from spleen into other organs was significantly attenuated, suggesting a noticeable immunomodulatory and anti-inflammatory action of the frankincense extract.

Lipopolysaccharides-only-treated animals also showed adrenal hypertrophy, which may be explained by activation of the hypothalamic–pituitary–adrenocortical axis in response to the inflammatory event (Prigent et al., 2004; Yang et al., 2007; Zhang et al., 2014), but also by stimulation of adrenal medullary catecholamine synthesis to stabilize cardiovascular function (Flierl et al., 2008). Probably by decreasing pro-inflammatory cytokine levels, thus exerting anti-inflammatory effects, Casp was evidently able to counteract these effects, since adrenal weights were significantly returned back to normal values in our investigation.

In contrast to spleen and adrenals, thymus weights were distinctly reduced after LPS treatment. As in spleen, this effect may be due to a massive emigration of T-lymphocytes into other organs, but also to increased apoptotic processes as has been shown in other sepsis animal experiments before (Hiramatsu et al., 1997, Seemann and Lupp, 2015). There was, however, also a comparable decrease in thymus weights and also a reduction in spleen weights after sole soy lecithin or Casp treatment. These effects remain unclear since no overt histological changes were noticed in the histological stainings of both organs after sole soy lecithin or Casp administration. The effects of Casp on the weights of these two immune organs could be, however, an expression of the known immunomodulatory properties of the boswellic acids (see e.g., Poeckel and Werz, 2006; Moussaieff and Mechoulam, 2009; Abdel-Tawab et al., 2011; Stürner et al., 2017), which in our mouse model evidently helped the animals to better cope with the LPS-induced systemic inflammation. After combined treatment with LPS, clear-cut protective effects were seen on all parameters tested, including spleen weights, plasma levels of pro- and anti-inflammatory cytokines, and immune cell redistribution, and there was no additional negative influence on thymus weights. Nevertheless, in future experiments, the effects of Casp (but also of soy lecithin) on the immune system in systemic inflammation should be elaborated in more detail in order to elucidate the underlying mechanisms of the protective findings of the present investigation more precisely and to exclude any adverse effects.

Lipopolysaccharides can lead to enhanced generation of reactive oxygen (ROS) and nitrogen species (RNS), both directly and via increased pro-inflammatory cytokine expression. ROS are the result of elevated NADPH-oxidase expression in neutrophils and monocytes/macrophages after LPS challenge (Kim et al., 2007), but may also be caused by increased mitochondrial leakage (Shoji et al., 1995; Galley, 2011). RNSs are generated both via enhanced NADPH-oxidase and iNOS expression in neutrophils and monocytes/macrophages. ROS/RNS are neutralized, among others, by the glutathione system, which represents an important anti-oxidative mechanism of the body to protect cellular macromolecules such as DNA, lipids, and proteins from chemical damage and degradation (Hayes and McLellan, 1999). In the present investigation, a distinct decrease in glutathione content in livers was observed after LPS treatment of the animals. This glutathione loss may either be caused by LPS-induced liver cell damage, as indicated by increased serum ALAT values, and subsequent impairment of glutathione synthesis, but may also be caused by intensified export to other organs, such as kidneys, lungs, and spleens. This latter hypothesis is supported by the observation that in these organs, and in contrast to liver, an increase in glutathione content was observed after LPS treatment. Lipid peroxidation products are the result of excessive oxidative stress which has overwhelmed the overall anti-oxidative capacity of the body and has led to a degradation of membrane lipids (Ayala et al., 2014). In the present investigation, corresponding to elevated ALAT values, a strong increase in lipid peroxidation products was seen especially in livers after LPS treatment, but also in all other organs tested. Casp caused a marked reduction in hepatic LPO content when given in addition to LPS, presumably by direct binding to LPS, thereby preventing increased expression of NADPH-oxidase, iNOS, and pro-inflammatory cytokines, but probably also due to a direct radical scavenging capacity (Afsar et al., 2012; Al-Harrasi et al., 2013). In all other organs investigated, levels were even reverted back to normal values. Altogether, these effects suggest a noticeable anti-oxidative capacity of the frankincense extract.

Biotransformation capacity is of essential importance for the detoxification and elimination of endogenous substances, as well as exogenously administered therapies, and the CYP system represents one of the key enzyme systems involved in phase I metabolism of xenobiotics (Nebert and Russell, 2002). It is well known from the literature that the pro-inflammatory cytokines TNF-α, IL-1β, IL-6, interferon-α, and interferon-γ, as well as NO cause a strong downregulation especially of the CYP system but also of other biotransformation enzymes (Morgan, 2001; Aitken et al., 2006). Consequently, in sepsis patients CYP-mediated drug metabolism is markedly impaired (Carcillo et al., 2003; Jacob et al., 2009). Since sepsis patients usually are concomitantly treated with several drugs, such as, e.g., antibiotics, reduction in CYP activity may lead to an accumulation of these substances and enhanced side effects of these multiple medications, which may have additional negative impact on the health status of these patients (Carcillo et al., 2003). In the present investigation, Casp turned out to be a strong inducer of all CYP enzymes tested. Since no other comparable *in vivo* data are available so far for either the frankincense extract or individual boswellic acids, this effect remains to be further elucidated. In the only *in vitro* study performed so far on liver microsomes, an inhibitory action of frankincense extract and various boswellic acids on different CYP enzymes has been shown (Frank and Unger, 2006). However, *in vitro* experiments using microsomes do not allow for prediction of potential drug effects as CYP inducer or inhibitor *in vivo* beyond a mere interaction, since for CYP induction intact cells are required. Based on the CYP-inducing capacity and the decreased levels of pro-inflammatory cytokines and NO, we postulate that Casp may be able to restore both CYP enzymes expression and activities back to normal values, which would suggest a therapeutic use of frankincense extract in the treatment of sepsis patients.

Besides the distinct beneficial effects of Casp on most of the parameters tested, in some instances a significant protective influence on LPS-mediated changes was also observed with the phospholipid soy lecithin. Thus, soy lecithin was able to cause a slight reduction in elevated LPO levels in lungs, hearts, and spleens and an improvement of CYP-mediated biotransformation capacity in some of the model reactions. However, in all these cases, the effect of Casp (i.e., the combination of soy lecithin and frankincense extract) exceeded that of soy lecithin alone. Protective effects of phospholipid-rich lipid solutions in sepsis have been reported both in humans and in animal experiments (Read et al., 1995; Goldfarb et al., 2003; Gordon et al., 2005). The exact mechanism underlying these effects still remains to be elucidated. However, an increased binding of LPS by lipoproteins generated from these lipids and thus prevention of interaction of the LPS/LPS-binding protein complex with Toll-like receptors has been suggested (Read et al., 1995; Goldfarb et al., 2003; Gordon et al., 2005).

## Conclusion

In summary, the present investigation shows for the first time that the orally bioavailable frankincense extract Casp is able to prevent acute LPS-induced systemic inflammation in mice by exerting anti-inflammatory, anti-oxidative, and hepatoprotective effects. Casp may therefore serve as a new prophylactically acting supportive treatment option in systemic inflammatory conditions, especially when accompanied by impaired liver function. It would be of further interest to determine whether Casp also exerts protective effects when given later in the course of the disease and/or using other sepsis models. Additionally, individual boswellic acids should be tested to find out, which of the different components of frankincense extract may indeed be responsible for the beneficial effects of Casp in acute systemic inflammation.

## Availability of Data and Materials

The materials used and raw data supporting the conclusions of the present manuscript will be available from the corresponding author without undue reservation on reasonable request to any qualified researcher.

## Author Contributions

KL, OW, and AL conceived and designed the experiments. MA-T and OW provided the compounds. AK, OW, and AL supervised the experiments. KL, SS, IL, SK, and AL performed the experiments. KL, SK, and AL analyzed the data. AL interpreted the data. AL wrote the manuscript. KL, SS, IL, SK, MA-T, AK, OW, and AL critically revised the manuscript. KL, SS, IL, SK, MA-T, AK, OW, and AL gave the final approval to the version to be published. All authors have read and approved the final version of the manuscript. Each of the authors acknowledges that he or she participated sufficiently in the work to take public responsibility for its content and each of the authors agreed to be accountable for all aspects of the work in ensuring that questions related to the accuracy or integrity of any part of the work are appropriately investigated and resolved.

## Conflict of Interest Statement

The authors declare that the research was conducted in the absence of any commercial or financial relationships that could be construed as a potential conflict of interest.

## Abbreviations

ALAT: alanine aminotransferase
BROD: benzyloxyresorufin-*O*-debenzylation
b.wt.: body weight
Casp: Casperome^®^
CD: cluster of differentiation
COX: cyclooxygenase
CSS: clinical severity score
CYP: cytochrome P450
ECOD: ethoxycoumarin-*O*-deethylation
EMND: ethylmorphine-*N*-demethylation
EROD: ethoxyresorufin-*O*-deethylation
GSH: reduced glutathione
GSSG: oxidized glutathione
IL: interleukin
iNOS: inducible nitric oxide synthase
LPO: lipid peroxides
LPS: lipopolysaccharides
MROD: methoxyresorufin-*O*-demethylation
NO: nitric oxide
Phyto: soy lecithin
PROD: pentoxyresorufin-*O*-depentylation
TBARS: thiobarbituric acid reactive substances
TNF: tumor necrosis factor
